# Daphnetin Alleviates Senile and Disuse Osteoporosis by Distinct Modulations of Bone Formation and Resorption

**DOI:** 10.3390/antiox11122365

**Published:** 2022-11-29

**Authors:** Jing Gao, Zhen Wang, Peipei Gao, Qiang Fan, Tiantian Zhang, Li Cui, Liujia Shi, Zhongbo Liu, Zhiwei Yang, Langchong He, Chunyan Wang, Yinghui Li, Lina Qu, Jiankang Liu, Jiangang Long

**Affiliations:** 1 Center for Mitochondrial Biology and Medicine, The Key Laboratory of Biomedical Information Engineering of Ministry of Education, School of Life Science and Technology, Xi’an Jiaotong University, Xi’an 710049, China; 2 Department of Cellular and Molecular Biology, State Key Laboratory of Space Medicine Fundamentals and Application, China Astronaut Research and Training Center, Beijing 100094, China; 3 Key Laboratory of Shaanxi Province for Craniofacial Precision Medicine Research, Laboratory Center of Stomatology, College of Stomatology, Xi’an Jiaotong University, Xi’an 710004, China; 4 School of Physics, Xi’an Jiaotong University, Xi’an 710049, China; 5 School of Pharmacy, Xi’an Jiaotong University, Xi’an 710061, China; 6 School of Health and Life Sciences, University of Health and Rehabilitation Sciences, Qingdao 266071, China

**Keywords:** daphnetin, senile osteoporosis, disuse osteoporosis, osteoblast, osteoclast, NADPH oxidase, reactive oxygen species (ROS), mitochondrial homeostasis

## Abstract

Senile and disuse osteoporosis have distinct bone turnover status and lack effective treatments. In this study, senescence-accelerated mouse prone 8 (SAMP8) and hindlimb unloading mouse models were used to explore the protective effects of daphnetin on these two types of osteoporosis, and primary osteoblasts and bone marrow monocyte-derived osteoclasts, as well as pre-osteoblast MC3T3-E1, and osteoclast precursor RAW264.7 cells were used to investigate the underlying mechanisms. The results showed that daphnetin administration effectively improved bone remodeling in both senile and disuse osteoporosis, but with different mechanisms. In senile osteoporosis with low bone turnover, daphnetin inhibited NOX2-mediated ROS production in osteoblasts, resulting in accelerated osteogenic differentiation and bone formation, while in disuse osteoporosis with high bone turnover, daphnetin restored SIRT3 expression, maintained mitochondrial homeostasis, and additionally upregulated SOD2 to eliminate ROS in osteoclasts, resulting in attenuation of osteoclast differentiation and bone resorption. These findings illuminated that daphnetin has promising potential for the prevention and treatment of senile and disuse osteoporosis. The different mechanisms may provide clues and basis for targeted prevention and treatment of osteoporosis according to distinct bone turnover status.

## 1. Introduction

Skeletal quality and integrity are maintained by a reconstruction process, which is balanced between osteoblastic bone formation and osteoclastic bone resorption [1]. If the rate of bone resorption is higher than that of bone formation, the volume and mineral density of bone will decrease, resulting in deterioration of bone microarchitecture and weakened mechanical strength such as hardness and elasticity of bone [2]. As a worldwide common skeletal disease, osteoporosis also causes fractures, physical pain, and disability, seriously affecting the life quality of patients [3]. Human osteoporosis mainly includes senile osteoporosis, postmenopausal osteoporosis, and glucocorticoid-induced osteoporosis. Besides these, weightlessness and other metabolic diseases such as type 2 diabetes are also important risk factors for osteoporosis [4]. With the accelerated aging of the population all over the world, the prevalence of senile osteoporosis is increasing rapidly [5]. In addition, disuse osteoporosis caused by bedridden immobilization, spinal cord injury, and space flight can also induce local or systemic reduction of bone mass [6]. Prevention and treatment of senile and disuse osteoporosis are not only medical problems worldwide, but also important challenges for the development of the aerospace industry.

At present, osteoporosis is generally prevented by dietary supplementation of calcium and vitamin D, as well as increasing exercise appropriately [7]. However, these strategies usually take a long time to produce the desired results, and the effects are also limited. Furthermore, exercise is not applicable for disuse osteoporosis. Excessive exercise may even have certain side effects on the bones [8]. In clinical studies to date, the medical treatments of osteoporosis have similarly exhibited limited curative effects and serious side effects [9,10,11,12]. Therefore, there is still a lack of therapeutic measures available to safely and effectively prevent or control the progression of osteoporosis.

Daphnetin (7,8-dihydroxycoumarin) is a bioactive molecule isolated from the plant of *Daphne*, a genus of deciduous and evergreen shrubs in the family Thymelaeaceae. As a widely studied natural compound, daphnetin has been proven to have various pharmacological activities such as antiinflammation [13], antioxidation [14], neuroprotection [15], and cardiovascular protections [16]. Previous studies have shown that daphnetin inhibited receptor activator of nuclear factor κB ligand (RANKL)-induced osteoclast formation in vitro through inhibiting reactive oxygen species (ROS) signal transduction, as well as preventing the activation of nuclear factor-kappa B (NF-Κb) and protein kinase B (Akt)/glycogen synthase kinase-3β (GSK-3β) signaling pathways [17]; ameliorated lipopolysaccharide (LPS)-induced inflammatory osteolysis and RANKL-induced osteoclastogenesis through inhibiting the extracellular signal-regulated kinase (ERK) and nuclear factor of activated T cells 1 (NFATc1) pathways [18], and played protective role in glucocorticoid-induced osteoporosis through activating the Wnt/GSK-3β/β-catenin signaling pathway [19]. However, the effects of daphnetin on senile and disuse osteoporosis remain unexplored.

In this study, we investigated the effects of daphnetin on senile and disuse osteoporosis as well as the possible involved mechanisms through in vivo and in vitro experiments. In terms of in vivo experiments, the classic senescence-accelerated mouse prone 8 (SAMP8) model [20] and hindlimb-unloading mouse model [21], which correspond to osteoporosis in elderly people, bedridden patients, and astronauts, were used to investigate the effects of daphnetin on senile and disuse osteoporosis. To further explore the possible mechanisms underlying the osteoprotective effects of daphnetin, in vitro experiments were performed with primary osteoblasts and bone marrow monocyte-derived osteoclasts, as well as osteoblast precursor MC3T3-E1 cells and osteoclast precursor RAW264.7 cells.

## 2. Materials and Methods

### 2.1. Reagents

Daphnetin (CAS#486-35-1, purity HPLC 98%) was purchased from Nanjing Xinhou Biotechnology Co., Ltd. (Nanjing, China) Minimum Essential Medium α (α-MEM), Trypsin, penicillin, and streptomycin were obtained from Gibco Laboratories (Life Technologies, Inc., Burlington, ON, Canada). Fetal bovine serum (FBS) was obtained from Biological Industries (BioInd, Kibbutz Beit Haemek, Israel). TriPure Isolation Reagent was purchased from Roche (Basel, Switzerland). Antibodies against manganese superoxide dismutase (SOD2), catalase (CAT), heme oxygenase-1 (HO-1), NADPH: quinone oxidoreductase 1 (NQO1), and nuclear respiratory factor 1 (Nrf1) were purchased from Santa Cruz Biotechnology (Santa Cruz, CA, USA). Antibody against runt related transcription factor 2 (Runx2) was purchased from Abcam (Cambridge, UK). Antibodies against mitochondrial complexes I~V were purchased from Life Technologies (San Diego, CA, USA). Antibodies against optic atrophy 1 (OPA1) and dynamin-related protein 1 (DRP1) were purchased from BD Biosciences (Franklin Lakes, NJ, USA). Antibodies against osteocalcin (OCN), sirtuin 3 (SIRT3), NFATc1, and glyceraldehyde-3-phosphate dehydrogenase (GAPDH) were purchased from Cell Signaling Technology (Danvers, MA, USA). Peroxidase-conjugated AffiniPure goat anti-rabbit IgG (H + L) and peroxidase-conjugated AffiniPure rabbit anti-mouse IgG (H + L) antibodies were purchased from Jackson ImmunoResearch (West Grove, PA, USA). All other chemicals were of analytical grade and obtained from Sigma Aldrich (St. Louis, MO, USA).

### 2.2. Animals and Treatments

In terms of senile osteoporosis, three-month-old male SAMP8 mice and senescence-accelerated mouse resistance 1 (SAMR1) mice were obtained from the Experimental Animal Center, Peking University. After one week of adaptation, the mice were randomly divided into three groups: SAMR1 group (*n* = 9), SAMP8 group (*n* = 9), and SAMP8 + daphnetin group (*n* = 6). Mice in the daphnetin-treated SAMP8 group were gavaged with daphnetin (100 mg/kg/d) for 3 months, meanwhile, mice in the SAMR1 group and SAMP8 group were gavaged with phosphate-buffered saline (PBS) followed by 4 months of normal feeding. The daphnetin dose and treatment time were chosen based on our preliminary test. Additionally, this dose has been proved to be effective and non-toxic by other in vivo studies [22,23].

In terms of disuse osteoporosis, two-month-old male C57BL/6 mice were obtained from Beijing Vital River Laboratory Animal Technology Co., Ltd. (Beijing, China). After one week of adaptation, the mice were randomly divided into three groups: control group (*n* = 9), unloading group (*n* = 9), and unloading + daphnetin group (*n* = 9). Mice in the daphnetin-treated unloading group were gavaged with daphnetin (100 mg/kg/d) for 7 days prior to tail suspension and continued with daphnetin gavage for 28 days after tail suspension. Meanwhile, mice in the control group and unloading group were gavaged with PBS.

All animals were housed in a temperature (25~28 °C)- and humidity (60%)-controlled animal room and maintained on a 12 h light/12 h dark cycle with free access to food and water during experiments. Animal procedures were approved by Xi’an Jiaotong University Animal Care and Use Committee (Approval No.: XJTU-2019-21). All animal studies complied with the ARRIVE guidelines, and all efforts were made to minimize the stress and the number of animals used in this study.

### 2.3. Cell Culture and In Vitro Differentiation

Primary osteoblasts were obtained by enzyme digestion of hindlimb bones from mice following a published protocol with minor modifications [24]. Briefly, hindlimb bones were isolated from the sacrificed mice and washed with PBS supplemented with 100 U/mL penicillin and 100 μg/mL streptomycin. After removing the soft tissues and bone marrow, the long bones were cut into pieces and digested with 2 mg/mL collagenase II in the culture medium at 37 °C for 2 h with gentle shaking every 5 min. Digestion solution was changed every 30 min. After digestion, the bone chips were washed and suspended in complete cell culture medium, followed by static culture for 11~15 days. Primary osteoblasts growing out of the bone chips were trypsinized and transferred into new dishes or plates for further experiments. When primary osteoblasts reached 90~100% confluence, osteogenic differentiation was initiated with 10 mM β-glycerophosphate and 50 μg/mL ascorbic acid for the indicated time periods.

Bone marrow monocyte-derived osteoclasts were isolated from the marrow of hindlimb bones following a published protocol with minor modifications [24]. Briefly, hindlimb bones were isolated from the sacrificed mice and washed with PBS. After scraping away the soft tissue, epiphyses were cut off and the bone marrow was flushed out with complete cell culture medium using a 25-gauge needle. The bone marrow was collected in a 50-mL centrifuge tube, and the cells were precipitated by spinning at 300 g for 5 min at room temperature. Cell pellet was resuspended in complete cell culture medium containing 40 ng/mL macrophage colony stimulating factor 1 (M-CSF) (Peprotech, Rocky Hill, NJ, USA) and cultured for 72 h. Then 100 ng/mL RANKL (Peprotech, Rocky Hill, NJ, USA) was added to the medium to induce osteoclastic differentiation, and osteoclasts were acquired after differentiation for 3~4 days.

Primary osteoblasts and bone marrow monocyte-derived osteoclasts, together with murine osteoblast precursor MC3T3-E1 cells and osteoclast precursor RAW264.7 cells purchased from National Collection of Authenticated Cell Cultures (NCACC, Shanghai, China), were all grown in α-MEM supplemented with 10% (*v*/*v*) FBS, 0.22% sodium bicarbonate, 100 U/mL penicillin, and 100 μg/mL streptomycin at 37 °C in a humidified 5% CO_2_ atmosphere. When MC3T3-E1 cells reached 90~100% confluence, osteogenic differentiation was initiated with 10 mM β-glycerophosphate and 50 μg/mL ascorbic acid, together with 0, 0.1, 0.5, and 1 μM daphnetin treatments for the indicated periods. When RAW264.7 cells reached 60% confluence, osteoclast differentiation was initiated with 100 ng/mL RANKL, together with 0, 0.1, 0.5, and 1 μM daphnetin treatments for the indicated periods. Differentiation medium was changed every 2~3 days.

### 2.4. Micro-CT Analysis of Bone Structure

To determine the bone mineral density (BMD) and microarchitecture of bone, the left femurs of mice were scanned using a micro-CT scanner (Quantum GX, PerkinElmer, Antwerp, Belgium). After reconstruction, the micro-CT data were loaded into Analyze 12.0 software (AnalyzeDirect, Overland Park, KS, USA) for calculation. For cancellous bone analysis, trabecular BMD, bone volume fraction (BV/TV), bone surface/bone volume (BS/BV), trabecular thickness (Tb. Th), trabecular separation (Tb. Sp), and connection density were calculated from a 250-slice round region of the distal femur. For cortical bone analysis, cortical BMD, BV/TV, BS/BV, cortical bone thickness (Ct. Th), and cortical area fraction (Ct. Ar/Tt. Ar) were calculated from a 60-slice round region of the mid-diaphysis femur.

### 2.5. Three-Point Bending Test

To evaluate bone biomechanical properties, the right femurs of sacrificed mice were removed and cleaned of soft tissues. Then three-point bending test was performed using a high precision material test system Instron Microtester 5848 (Instron, Norwood, MA, USA). The Instron machine was set to a loading rate of 1.2 mm/min. Each femur was placed in the same orientation on supports placed 1 cm apart. The Loading-Displacement curve was recorded as the ultimate breaking force required to break the bone, and the ultimate force, bending energy absorption and elastic modulus were calculated.

### 2.6. Hydroxyproline Determination

To evaluate collagen metabolism in bone tissues, hydroxyproline as the main component of collagen was measured with Hydroxyproline assay kit (Lot No.: A030-2-1, Nanjing Jiancheng Bioengineering Institute, Nanjing, China) according to manufacturer’s instructions. The content of hydroxyproline was calculated according to OD550 nm measured with a spectrometer (FlexStation 3, Molecular Devices, Sunnyvale, CA, USA).

### 2.7. Bone Tissue Section Staining

Femur tissues dissected from mice were fixed with 10% formalin for 48 h and decalcified in 14% (*w*/*v*) ethylenediaminetetraacetic acid (EDTA) in PBS (pH 7.4) for 21 days at room temperature. Sections were cut from paraffin wax embedded tissues, then, hematoxylin-eosin (H & E) staining was performed to observe the pathomorphological changes of bone tissues. Alizarin Red S (ARS) staining was performed using Alizarin Red S solution (1%, pH 4.2) (Solarbio, Beijing, China) to detect the matrix mineralization deposition of osteoblasts. Tartrate resistant acid phosphatase (TRAP) staining was performed using Acid Phosphatase, Leukocyte (TRAP) Kit (Sigma-Aldrich, St. Louis, MO, USA) to evaluate osteoclastogenesis.

### 2.8. Determination of Bone Turnover Parameters

Blood samples were allowed to clot undisturbed for 30 min at room temperature. Serum was separated through centrifugation at 3000× *g* for 10 min. Bone tissues were homogenized with the aid of a Polytron homogenizer in ice-cold PBS, and bone homogenates were prepared through centrifugation at 4 °C, 1000× *g* for 10 min. Both serum and bone homogenate were used for subsequent analysis. The levels of biochemical markers of bone turnover such as bone alkaline phosphatase (BAP), bone Gla-protein (BGP), osteoprotegerin (OPG), procollagen type I N-terminal propeptide (P1NP), β isomer of C-terminal telopeptide of type I collagen (β-CTX), RANKL, and tartrate-resistant acid phosphatase 5b (TRACP-5b) were measured by enzyme-linked immunosorbent assay (ELISA).

### 2.9. Alkaline Phosphatase (ALP) Activity Assay

ALP activity of bone tissue and osteoblast homogenate was measured with a commercially available assay kit (Lot No.: A059-2, Nanjing Jiancheng Bioengineering Institute) according to manufacturer’s instructions. ALP activity was calculated according to OD520 nm measured with spectrometer (FlexStation 3, Molecular Devices, Sunnyvale, CA, USA).

### 2.10. TRAP Activity Assay

TRAP activity of bone tissue and osteoclast homogenates was measured with a commercially available assay kit (Lot No.: TE0043, Leagene Biotechnology, Inc., Beijing, China) according to manufacturer’s instructions. The activity of TRAP was calculated according to OD 400~415 nm measured with spectrometer (FlexStation 3, Molecular Devices, Sunnyvale, CA, USA).

### 2.11. ALP Staining of Osteoblasts

Primary osteoblasts isolated from SAMP8 mice were induced to differentiate for 7 days, then the intracellular ALP level was determined by ALP staining using BCIP/NBT alkaline phosphatase color development kit (Lot No.: C3206, Beyotime, Haimen, China) according to the manufacturer’s instructions, then the stained matrix was observed with light microscope.

### 2.12. Alizarin Red S (ARS) Staining of Osteoblasts

Primary osteoblasts isolated from SAMP8 mice, and MC3T3-E1 cells were induced to differentiate for 7 or 14 days, then mineralization of cell matrix was determined by the deposition of crystalline hydroxyapatite and visualized by ARS staining. Cells were gently washed twice with PBS and fixed with ice-cold 70% (*v*/*v*) ethanol for 30 min at 4 °C. Then, cells were washed with PBS for three times, and stained with 5% ARS solution (pH 4.2) for 30 min at 37 °C. Cells were finally washed with PBS to remove excess dye, and the stained matrix was photographed and observed with light microscope.

### 2.13. RNA Isolation and Quantitative Reverse Transcriptase-Polymerase Chain Reaction (qRT-PCR)

After various treatments, cells were lysed using TRIzol reagent, then total RNA was separated by chloroform, precipitated by isopropanol, and washed with 75% (*v*/*v*) ethanol. After quantification, reverse transcription was performed using the PrimeScript RT-PCR Kit (Lot No.: RR036A, Takara, Otsu, Shiga, Japan) followed by quantitative real-time PCR using real-time PCR Master Mix (Lot No.: RR820A, Takara, Otsu, Shiga, Japan) following the manufacturer’s protocol. Melting curves were assessed over the range 55~99 °C to ensure specific DNA amplification. The cycle number at which the various transcripts were detected (Ct) was compared with that of GAPDH, referred to as ΔCt. Results were presented as a percentage of the control. Sequences of primers used in the real-time PCR were listed in Table S1. The primers were derived from GenBank, verified by Primer-BLAST of NCBI, and synthesized by Qingke Biotechnology Co., Ltd. (Beijing, China).

### 2.14. Protein Extraction and Western Blot Analysis

After various treatments, cells were washed twice with ice-cold PBS and lysed with Western and IP lysis buffer (Beyotime, Haimen, China) containing 1 mM phenylmethanesulfonylfluoride on ice. The lysates were centrifuged at 13,000× *g* for 6 min at 4 °C. Protein concentrations of the collected supernatants were quantified with BCA kit (Lot No.: 23225, Pierce Biotechnology, Rockford, IL, USA). Equal aliquots of protein samples were subjected to SDS-PAGE, and then transferred to NC membranes. After blocking with 5% (*w*/*v*) skimmed milk for 1 h at room temperature, membranes were incubated with primary antibodies (1:1000 dilution) at 4 °C overnight. After washing with TBST three times (15 min each), the membranes were incubated with horseradish-peroxidase-conjugated secondary antibodies (1:4000 dilution) for 1 h at room temperature. After washing with TBST three times, chemiluminescent detection was performed using ECL western blotting substrate (Bio-Rad, Hercules, CA, USA) and quantified by scanning densitometry.

### 2.15. Cell Viability Assay

MC3T3-E1 and RAW264.7 cells were seeded in 96-well plates. After being treated with indicated concentrations of daphnetin for 48 h, cells were incubated with 0.5 mg/mL 3-(4, 5-dimethylthiazol-2-yl)-2, 5-diphenyltetrazolium bromide (MTT) dissolved in FBS-free DMEM for 4 h at 37 °C. Then, the supernatants were removed, and MTT-formazan products were solubilized with 200 mL DMSO each well. The optical densities were measured at 550 nm using a microplate spectrophotometer (FlexStation 3, Molecular Devices, Sunnyvale, CA, USA).

### 2.16. Determination of Intracellular ROS

After various treatments, cells were incubated with 10 μM H_2_DCF-DA (Life Technologies, San Diego, CA, USA) in cell culture medium for 30 min at 37 °C and lysed with solution containing 10 mM Tris-base, 150 mM NaCl, 0.1 mM EDTA-Na_2_, and 0.5% (*v*/*v*) Triton X-100. Intracellular ROS level was determined by the formation of fluorescent 2′,7′-dichlorofluorescein upon oxidation of non-fluorescent, reduced DCFH [25]. The fluorescence intensity of the supernatant was measured with a fluorescence spectrometer (FlexStation 3, Molecular Devices, Sunnyvale, CA, USA) at 485 ex/538 em. Cellular oxidant levels were expressed as the relative fluorescence per microgram of protein.

### 2.17. Determination of Total Antioxidant Capacity (T-AOC)

T-AOC of cell homogenates was measured with a commercially available assay kit (Lot No.: A015, Nanjing Jiancheng Bioengineering Institute) according to the manufacturer’s instructions. T-AOC was calculated according to OD520 nm measured with a spectrometer (FlexStation 3, Molecular Devices, Sunnyvale, CA, USA).

### 2.18. Determination of GSH

GSH levels were measured with the 2,3-naphthalenedicarboxyaldehyde (NDA) method [26]. Briefly, 20 μL of cell homogenate and 180 μL of NDA derivatization solution [50 mM Tris (pH 10), 0.5 N NaOH and 10 mM NDA in Me2SO, *v*/*v*/*v* 1.4:0.2:0.2] were added to each well of a 96-well plate. The plate was covered to protect the wells from light and incubated at room temperature for 30 min. The NDA-GSH fluorescence intensity was measured (485 ex/538 em) with a fluorescence spectrometer (FlexStation 3, Molecular Devices, Sunnyvale, CA, USA).

### 2.19. Superoxide Dismutase 2 (SOD2) Activity Assay

SOD2 activity in cell homogenate was measured with a commercially available assay kit (Lot No.: A001-2-2, Nanjing Jiancheng Bioengineering Institute) according to the manufacturer’s instructions. Activities of total SOD and CuZn SOD were calculated according to OD550 nm measured with a spectrometer (FlexStation 3, Molecular Devices, Sunnyvale, CA, USA). SOD2 activity was equal to total SOD activity minus CuZn SOD activity.

### 2.20. Tartrate-Resistant Acid Phosphatase (TRAP) Staining of Osteoclasts

Bone marrow monocyte-derived osteoclasts isolated from hindlimb unloading mice, and RAW264.7 cells were induced to differentiate for 6 or 4 days, then TRAP staining was performed using Acid Phosphatase, Leukocyte (TRAP) Kit (Lot No.: 387A, Sigma-Aldrich, St. Louis, MO, USA) to evaluate osteoclastogenesis. Cells were gently washed twice with PBS and successively fixed with 10% (*v*/*v*) formaldehyde and fixative solution (ethanol: acetone 1:1) for 1 min at room temperature. Then, cells were washed with deionized water for three times, and stained with prewarmed staining solution (prepared according to the instructions) for 1 h at 37 °C protected from light. Cells were finally washed with deionized water to remove excess dye, and the stained matrix was photographed and observed with light microscope.

### 2.21. Statistical Analysis

The in vitro experiments were performed in triplicate and repeated at least three times. Data were presented as the mean ± S.E.M. and were analyzed using the GraphPad Prism version 8 software (San Diego, CA, USA). Statistical significance was evaluated using one-way analysis of variance (ANOVA) followed by Tukey’s HSD test. Value of *p* < 0.05 was considered as significant.

## 3. Results

### 3.1. Daphnetin Alleviated Senile Osteoporosis in SAMP8 Mice

**Figure 1:**
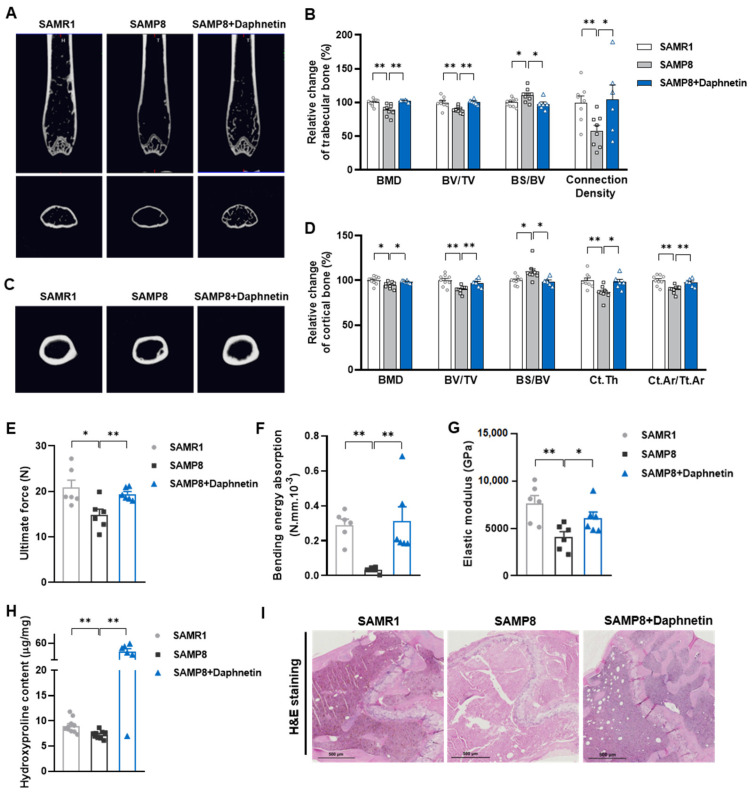
Protective effects of daphnetin against senile osteoporosis in SAMP8 mice. (**A**) Micro-CT images of trabecular bone in distal femur. (**B**) Trabecular bone mineral density (BMD), bone volume per tissue volume (BV/TV), bone surface to bone volume ratio (BS/BV), and connection density were calculated from the micro-CT data. (**C**) Micro-CT images of cortical bone in mid-diaphysis femur. (**D**) Cortical BMD, BV/TV, BS/BV, cortical thickness (Ct. Th), and cortical area to total area ratio (Ct. Ar/Tt. Ar) were calculated from the micro-CT data. To evaluate femoral mechanical properties, ultimate force (**E**), bending energy absorption (**F**), and elastic modulus (**G**) were determined by three-point bending test. (**H**) Hydroxyproline content of femur was determined with commercial kit. (**I**) Femur sections were prepared, and H&E staining was performed. Data are mean ± S.E.M. (*n* ≥ 6); * *p* < 0.05; ** *p* < 0.01.

To investigate the effects of daphnetin on senile osteoporosis, micro-CT analysis was performed in SAMR1, SAMP8, and daphnetin-treated SAMP8 mice. The results clearly show that compared with the control SAMR1 mice, SAMP8 mice exhibited obvious degenerative changes of trabecular mineral density and microstructure, indicated by lower BMD, BV/TV, connection density, and higher BS/BV. Daphnetin administration significantly reversed these changes (Figure 1A,B). Results in Figure 1C,D show that SAMP8 mice also exhibited lower BMD, BV/TV, Ct. Th, Ct. Ar/Tt. Ar and higher BS/BV in cortical bone, while daphnetin administration effectively protected SAMP8 mice from aging-induced cortical bone loss. Three-point bending test was also performed to evaluate the femoral mechanical properties of bone. The calculation results of ultimate force, bending energy absorption, and elastic modulus show significantly weakened bone strength in SAMP8 mice, while daphnetin administration effectively enhanced the hardness and toughness of bone (Figure 1E–G). Result in Figure 1H shows that the level of collagen’s main component hydroxyproline was also significantly increased in daphnetin-treated SAMP8 mice, indicating consistently improved collagen metabolism. Histomorphometry analysis by H&E staining shows that there were lesser bone trabeculae and thinner cortical bones in the distal femoral bone of SAMP8 mice than SAMR1 mice. In addition, increased bone volume and improved bone microstructure were observed in daphnetin-treated SAMP8 mice (Figure 1I).

### 3.2. Daphnetin Alleviated Disuse Osteoporosis in Hindlimb Unloading Mice

**Figure 2:**
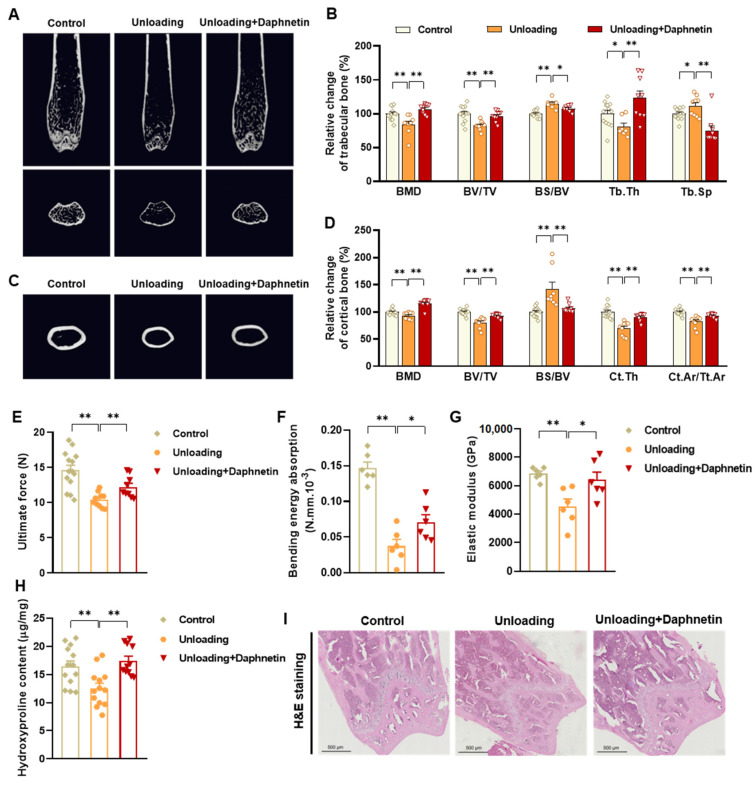
Protective effects of daphnetin against disuse osteoporosis in hindlimb unloading mice. (**A**) Micro-CT images of trabecular bone in distal femur. (**B**) Trabecular BMD, BV/TV, BS/BV, trabecular thickness (Tb. Th) and trabecular spacing (Tb. Sp) were calculated from the micro-CT data. (**C**) Micro-CT images of cortical bone in mid-diaphysis femur. (**D**) Cortical BMD, BV/TV, BS/BV, Ct.Th, and Ct. Ar/Tt. Ar were calculated from the micro-CT data. To evaluate femoral mechanical properties, ultimate force (**E**), bending energy absorption (**F**), and elastic modulus (**G**) were determined by three-point bending test. (**H**) Hydroxyproline content of femur was determined with commercial kit. (**I**) Femur sections were prepared, and H & E staining was performed. Data are mean ± S.E.M. (*n* ≥ 6); * *p* < 0.05; ** *p* < 0.01.

To investigate the potential role of daphnetin in disuse osteoporosis, similar experiments were performed on hindlimb unloading mice. Results of micro-CT analysis show that hindlimb unloading mice exhibited obvious degenerative changes of mineral density and microstructure of trabecular bone, indicated by lower BMD, BV/TV, Tb. Th and higher BS/BV, Tb. Sp, and daphnetin administration significantly reversed these changes (Figure 2A,B). Consistently, results in Figure 2C,D show that hindlimb unloading mice also exhibited lower BMD, BV/TV, Ct. Th, Ct. Ar/Tt. Ar, and higher BS/BV of cortical bone, which were also effectively reversed by daphnetin administration. To investigate the effect of daphnetin on bone strength of hindlimb unloading mice, three-point bending test was successively performed, and the results show that daphnetin significantly enhanced ultimate force, bending energy absorption and elastic modulus under disuse conditions (Figure 2E–G). The hydroxyproline content of bone tissue decreased significantly in hindlimb unloading mice, while daphnetin administration also effectively restored the collagen level (Figure 2H). Histomorphometry analysis by H & E staining shows that the distal femoral bones of unloading group had lesser bone trabeculae and thinner cortical bones than that of the control group, while daphnetin-treated unloading group exhibited significantly increased bone volume and improved bone microstructure (Figure 2I). Taken together, results in Section 3.1 and Section 3.2 indicate that daphnetin can alleviate both senile osteoporosis and disuse osteoporosis.

### 3.3. Distinct Effects of Daphnetin on Bone Remodeling of Senile and Disuse Osteoporosis with Different Bone Turnover Status

**Figure 3:**
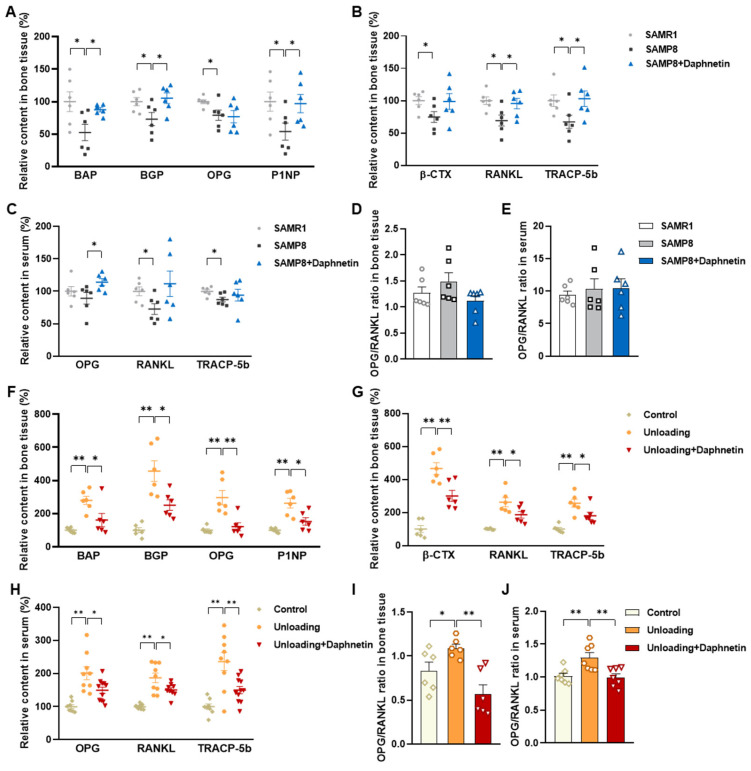
Effects of daphnetin on bone turnover status and OPG/RANKL pathway in SAMP8 and hindlimb unloading mice. In SAMP8 mice, the relative content of bone formation-related parameters (**A**), bone resorption-related parameters (**B**), and OPG/RANKL ratio in bone homogenates (**D**) were determined by ELISA. Serum levels of OPG, RANKL, and TRACP-5b (**C**), as well as OPG/RANKL ratio in serum (**E**) were also determined. In hindlimb unloading mice, the relative content of bone formation-related parameters (**F**), bone resorption-related parameters (**G**), and OPG/RANKL ratio in bone homogenates (**I**) were determined by ELISA. Serum levels of OPG, RANKL, and TRACP-5b (**H**), as well as OPG/RANKL ratio in serum (**J**) were also determined. Data are mean ± S.E.M. (*n* ≥ 6); * *p* < 0.05; ** *p* < 0.01.

To evaluate the effects of daphnetin on bone remodeling of SAMP8 and hindlimb unloading mice, the tissue contents and serum levels of characteristic parameters related to bone formation and bone resorption were determined. As is shown in Figure 3A,B, the tissue content of bone alkaline phosphatase (BAP), the bone-specific isoform of alkaline phosphatase, and a by-product of osteoblast activity were found to be significantly decreased in SAMP8 mice, accompanied with the decreased content of bone Gla-protein (BGP), a noncollagenous protein solely secreted by osteoblasts, as well as bone formation related biomarker OPG and procollagen type 1 amino-terminal propeptide (PINP). Meanwhile, the tissue content of β-isomerized C-telopeptide (β-CTX), which is released during bone resorption by osteoclasts, osteoclastogenesis inducer RANKL and osteoclastic marker enzyme tartrate-resistant acid phosphatase 5b (TRACP-5b) were also decreased in SAMP8 mice. In addition, the serum levels of OPG, RANKL, and TRACP-5b were consistent with markers in bone tissue (Figure 3C), indicating a slow bone turnover status in senile osteoporosis. On the contrary, the tissue content and serum levels of identical parameters related to bone formation and bone resorption all increased in hindlimb unloading mice (Figure 3F–H), indicating a high bone turnover status in disuse osteoporosis. However, no matter what existed in the bone turnover status, daphnetin administration almost reversed these changes. At the same time, the ratio of OPG/RANKL in bone tissue and serum were calculated, and there were no significant changes of OPG/RANKL in SAMP8 mice with or without administration of daphnetin (Figure 3D,E). Figure 3I,J show a significant increase of OPG/RANKL in hindlimb unloading mice, and daphnetin administration effectively made it return to normal.

**Figure 4:**
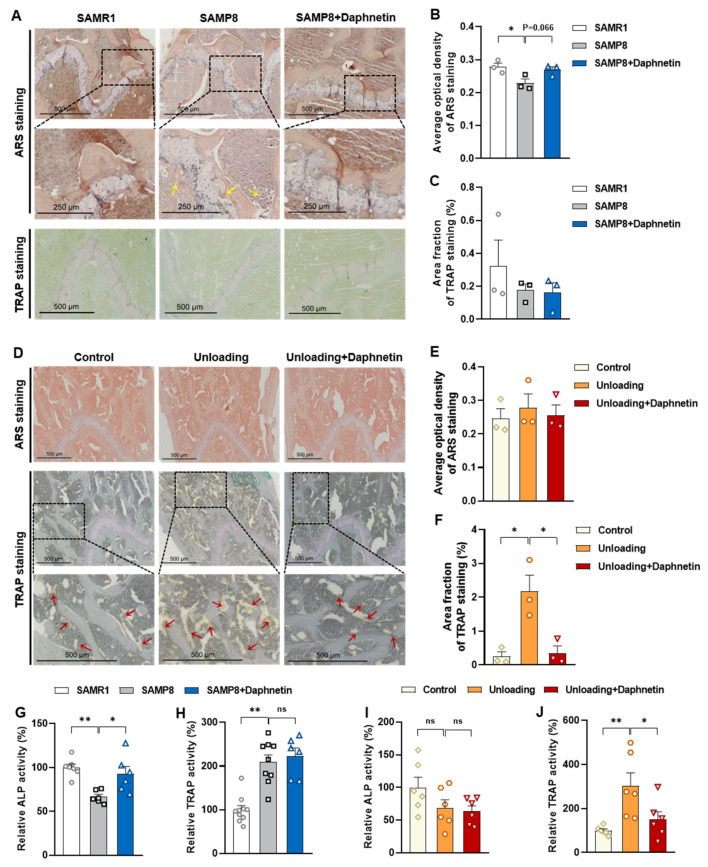
Histological observation and activity assay of bone formation and resorption in SAMP8 and hindlimb unloading mice with daphnetin administration. ARS staining and TRAP staining were performed on femur sections. (**A**) Images of ARS and TRAP staining of SAMP8 mice. (**D**) Images of ARS and TRAP staining of hindlimb unloading mice. (**B**,**E**) Statistical analysis of ARS staining was performed with ImageJ software (*n* = 3). (**C**,**F**) Statistical analysis of TRAP staining was performed with Image-Pro Plus software (*n* = 3). In SAMP8 mice, ALP activity (**G**) and TRAP activity (**H**) of bone homogenates were determined with commercial kits. In hindlimb unloading mice, ALP activity (**I**) and TRAP activity (**J**) of bone homogenates were also determined. Data are mean ± S.E.M. (*n* ≥ 6); * *p* < 0.05; ** *p* < 0.01; ns, not significant.

Osteoblastic ARS staining and osteoclastic TRAP staining were performed on femur sections of mice. In SAMP8 mice, the mineral deposition was significantly decreased, which tended to be reversed by daphnetin administration, while there were few significant changes of the TRAP positive osteoclasts (Figure 4A–C). In hindlimb unloading mice, comparing with ARS staining, the results of TRAP staining exhibited more obvious variations among experimental groups. Unloading mice exhibited significantly increased number of TRAP positive osteoclasts, which was effectively prevented by daphnetin administration (Figure 4D–F). Analogously, activities of ALP and TRAP, which are the characteristic enzymes of bone formation and bone resorption, were also determined. We found that daphnetin administration effectively prevented the decline of ALP activity in SAMP8 mice (Figure 4G) but had no effect on the enhanced activity of bone resorption enzyme TRAP (Figure 4H). However, in hindlimb unloading mice, daphnetin significantly inhibited TRAP activity (Figure 4J), but had no effect on bone formation enzyme ALP (Figure 4I).

### 3.4. Daphnetin Preserving Bone Formation in SAMP8 Mice Was Accompanied with Inhibition of NADPH Oxidase (NOX) 2-Mediated ROS Production

**Figure 5:**
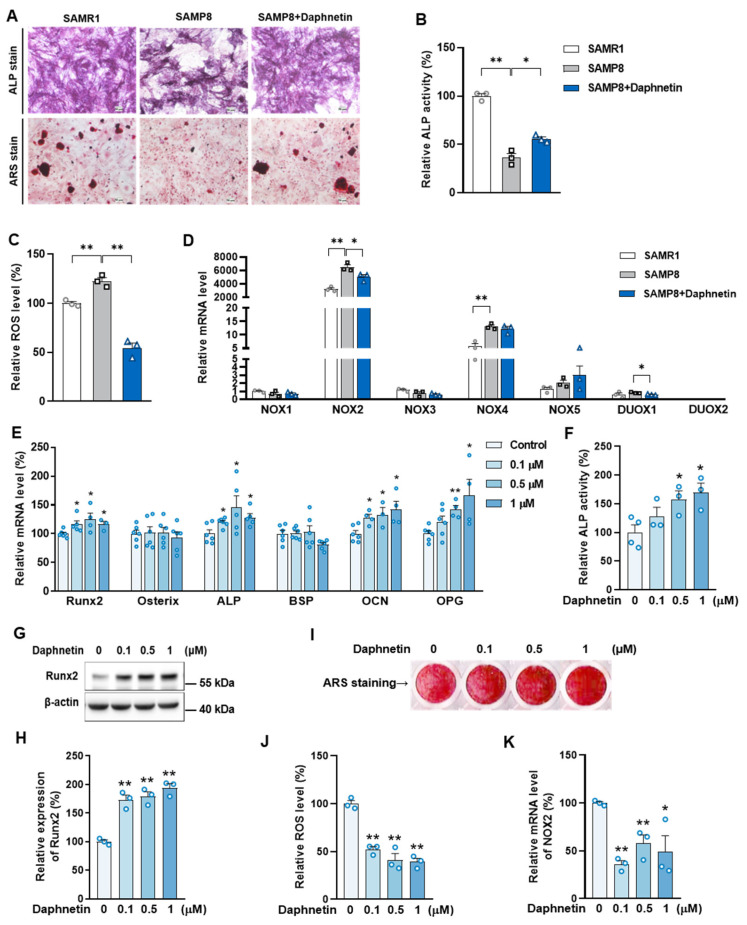
The decrease of ROS and inhibition of NOX2 in daphnetin-promoted osteogenesis. Primary osteoblasts were isolated from mice and induced differentiation in vitro. (**A**) ALP and ARS staining were performed and observed with microscope after differentiation for 7 days. (**B**) ALP activity in cell homogenate was determined after differentiation for 4 days. (**C**) Intracellular ROS level was determined with DCF probe after differentiation for 3 days. (**D**) The transcription of NOX family genes was determined by qRT-PCR after differentiation for 3 days. MC3T3-E1 cells treated with indicated concentrations of daphnetin were induced to differentiate in vitro. (**E**) The mRNA levels of bone formation-related marker genes were determined by qRT-PCR after differentiation for 2 days. (**F**) ALP activity in cell homogenates was determined after differentiation for 7 days. Expression of Runx2 was determined by Western blot ((**G**) Western blot images; (**H**) statistical analysis). (**I**) ARS staining was performed, and mineral deposition in cell matrix was photographed after differentiation for 14 days. (**J**) Intracellular ROS level was determined after daphnetin treatment for 24 h. (**K**) The mRNA level of NOX2 was determined by qRT-PCR after differentiation for 2 days. Data are mean ± S.E.M. (*n* ≥ 3); * *p* < 0.05; ** *p* < 0.01 between the connected groups or versus control.

To investigate the underlying mechanisms of the effects of daphnetin on senile osteoporosis, primary osteoblasts were isolated from SAMR1, SAMP8, and daphnetin-treated SAMP8 mice, and induced differentiation in vitro to evaluate their osteogenic differentiation capacity. As shown in Figure 5A, the significant decreased ALP content and mineralized deposition in osteoblasts of SAMP8 mice were effectively restored by daphnetin, indicating that daphnetin can preserve osteogenic differentiation of osteoblasts, which was hindered in senile osteoporosis. We examined ALP activity of cell homogenate, and the results were consistent with weakened osteogenic capacity of osteoblasts in SAMP8 mice (Figure 5B).

Mouse pre-osteoblast MC3T3-E1 cells were used to further evaluate the effects of daphnetin on bone formation under normal conditions. The concentrations of 0.1, 0.5, and 1 μM daphnetin were found non-cytotoxic by MTT assay and adopted in vitro experiments (Figure S1A). Consistent with the results of primary osteoblasts, after differentiation for two days with the indicated concentrations of daphnetin treatment, transcription of osteogenic marker genes such as Runx2, Osterix, ALP, bone sialoprotein (BSP), OCN, and OPG, expression of Runx2, as well as cellular ALP activity were significantly increased by daphnetin treatment (Figure 5E–H). After osteogenic differentiation for 14 days, the matrix mineralization deposition of MC3T3-E1 cells determined with ARS staining was also promoted by daphnetin in a dose-dependent manner (Figure 5I).

We also determined the intracellular ROS level of primary osteoblasts. There was an excessive ROS accumulation in osteoblasts of SAMP8 mice, while daphnetin administration significantly reduced aging-induced oxidative stress in osteoblasts (Figure 5C). Therefore, we explored the potential mechanisms of daphnetin against oxidative stress in osteoblasts of SAMP8 mice. Through evaluating the antioxidant capacity, we found that there were no significant changes in T-AOC, GSH level, SOD2 activity (Figure S2A–C), or expression of SOD2 (Figure S2D,E). Additionally, the decreased expression of antioxidant enzyme heme oxygenase 1 (HO-1) could not be restored by daphnetin, while the increased expression of catalase (CAT) and NAD (P) H: quinone oxidoreductase 1 (NQO1) as compensatory response to aging-induced oxidative stress, were reversed by daphnetin (Figure S2D,E). We further evaluated the expression of SIRT3 and mitochondria-related proteins and found that decreased expression of SIRT3 and abnormal changes of mitochondria-related proteins in SAMP8 mice were not reversed by daphnetin administration (Figure S2F–K).

Since phagocyte-like NADPH oxidase (NOX) as the homologs of catalytic subunit of the NOX is the major source of extra-mitochondrial ROS, the mRNA levels of NOX family genes were determined. As shown in Figure 5D, the transcription of both NOX2 and NOX4 were significantly activated in osteoblasts of SAMP8 mice. However, the increase of NOX2 was significantly higher than that of NOX4, and daphnetin administration only inhibited the increase of NOX2, but not NOX4. Therefore, we speculated that daphnetin may rescue osteogenesis of SAMP8 mice through inhibition of NOX2/ROS cascade. To confirm this speculation, the effects of daphnetin on NOX2/ROS cascade of MC3T3-E1 cells were investigated. As is shown in Figure 5J,K, daphnetin reduced intracellular ROS in a dose-dependent manner and significantly inhibited the transcription of NOX2 in MC3T3-E1 cells.

### 3.5. Daphnetin Attenuating Bone Resorption in Hindlimb Unloading Mice Was Accompanied with Restoration of SIRT3/SOD2 Pathway and Mitochondrial Homeostasis

**Figure 6:**
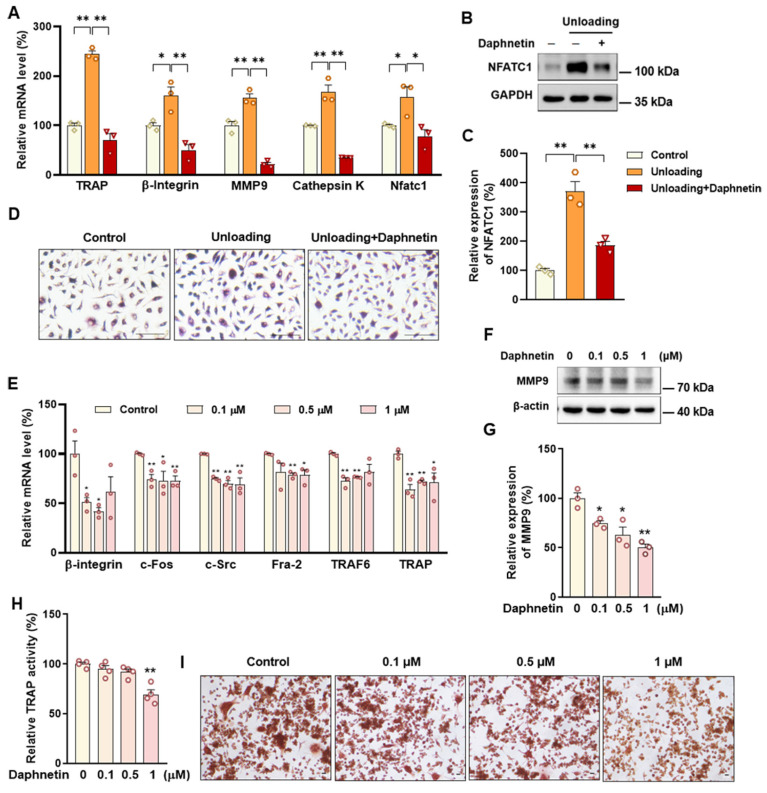
The effects of daphnetin on bone resorption in hindlimb unloading mice and osteoclast differentiation of RAW264.7 cells. Bone marrow monocyte-derived osteoclasts were also isolated from mice and induced to differentiate for 6 days. (**A**) The mRNA levels of bone resorption-related marker genes were determined by qRT-PCR. (**B**,**C**) The expression of NFATC1 was determined by Western blot (**B**: Western blot image; **C**: statistical analysis). (**D**) TRAP staining was performed and observed with microscope. RAW264.7 cells were treated with indicated concentrations of daphnetin, and induced differentiation in vitro. After differentiation for 3 days, the mRNA levels of bone resorption marker genes were determined by qRT-PCR (**E**), the expression of MMP9 was determined by Western blot ((**F**) Western blot image; (**G**) statistical analysis). (**H**) TRAP activity in cell homogenates was determined. (**I**) TRAP staining was performed and observed with microscope after differentiation for 4 days. Data are mean ± S.E.M. (*n* ≥ 3); * *p* < 0.05; ** *p* < 0.01 between the connected groups or versus control.

To further investigate the mechanisms underlying the protective effects of daphnetin on disuse osteoporosis, bone marrow monocyte-derived osteoclasts were isolated from control, hindlimb unloading, and daphnetin-treated unloading mice, and induced differentiation in vitro. In hindlimb unloading mice, the transcription of osteoclastic marker genes such as TRAP, β-Integrin, matrix metalloproteinase-9 (MMP9), cathepsin K, and Nfatc1 were significantly increased (Figure 6A), and the expression of NFATC1 was significantly up-regulated in osteoclasts (Figure 6B,C). Meanwhile, there was an enhanced staining of TRAP in bone marrow monocyte-derived osteoclasts of hindlimb unloading mice (Figure 6D), indicating increased formation and activity of osteoclasts. Daphnetin administration effectively reversed these changes (Figure 6A–D).

Osteoclast precursor RAW264.7 cells were used to further evaluate the effects of daphnetin on osteoclast differentiation under normal conditions. The adopted concentrations of daphnetin were also non-cytotoxic to RAW264.7 cells by MTT assay (Figure S1B). After osteoclastic differentiation for three days with the indicated concentrations of daphnetin treatment, transcriptions of osteoclastic marker genes such as β-integrin, c-Fos, c-Src, Fra-2, TNF receptor associated factor 6 (TRAF6), and TRAP were significantly decreased (Figure 6E), and expression of MMP9 was significantly down-regulated (Figure 6F,G). Activity of TRAP, a marker enzyme of osteoclasts, was significantly inhibited by 1 μM daphnetin treatment (Figure 6H). Meanwhile, daphnetin dose-dependently decreased the formation of numerous multinucleated TRAP positive osteoclasts (Figure 6I).

**Figure 7:**
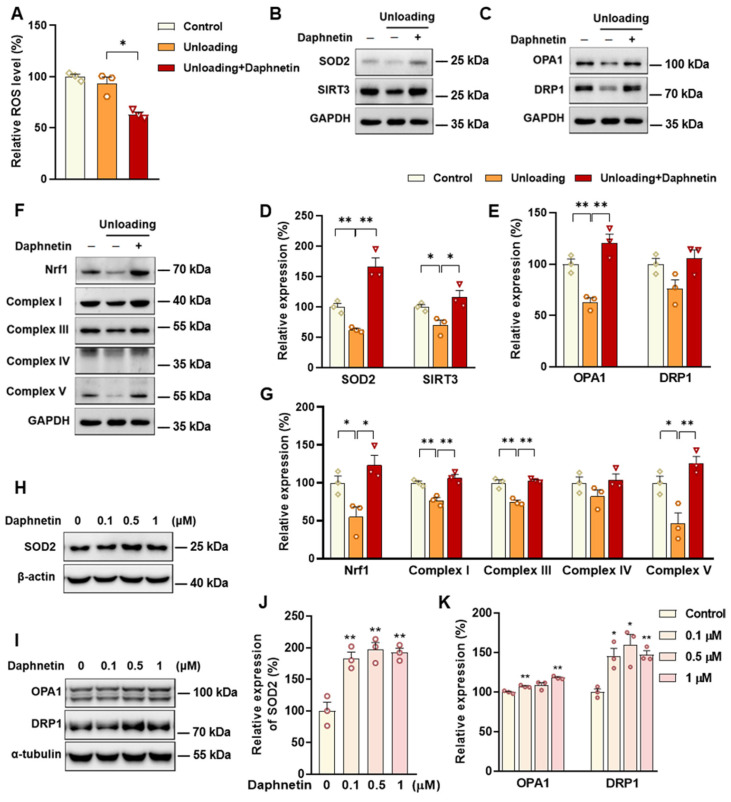
The effects of daphnetin on SIRT3/SOD2 pathway and mitochondrial homeostasis in osteoclastogenesis. Primary osteoblasts were isolated from mice and induced to differentiate for 2 days, (**A**) Intracellular ROS level in bone marrow monocyte-derived osteoclasts was determined with DCF probe. Expression of SOD2, SIRT3, mitochondrial dynamic- and biogenesis-related proteins were also determined by Western blot ((**B**,**C**,**F**) Western blot images; (**D**,**E**,**G**) statistical analysis). RAW264.7 cells treated with indicated concentrations of daphnetin were induced to differentiate in vitro. After differentiation for 3 days followed by daphnetin treatment for 6 h, expression of SOD2, OPA1 and DRP1 were determined by Western blot ((**H**,**I**) Western blot images; (**J**,**K**) statistical analysis). Data are mean ± S.E.M. (*n* = 3); * *p* < 0.05, ** *p* < 0.01 between the connected groups or versus control.

Daphnetin administration was found to cause a significant decrease in intracellular ROS in osteoclasts of hindlimb unloading mice (Figure 7A). To further explore the possible mechanisms underlying daphnetin-induced ROS elimination, we investigated the expressions of SOD2, SIRT3, and mitochondria-related proteins in osteoclasts. As shown in Figure 7B–G, the expression of SOD2 was significantly upregulated by daphnetin. In addition, the expressions of SIRT3, mitochondrial biogenesis-related proteins such as NRF1, mitochondrial complex I, III, IV, V, and mitochondrial-dynamic related proteins such as OPA1 and DRP1 were consistently decreased in osteoclast of unloading mice, while daphnetin administration almost reversed all of these abnormal changes. However, being different from the effect of daphnetin on osteoblasts under senile osteoporosis, there were no obvious changes in the transcription of NOX family genes (Figure S3A). The effects of daphnetin on SIRT3/SOD2 pathway and mitochondrial homeostasis of RAW264.7 cells were also investigated. Although under normal conditions, neither intracellular ROS nor expression of SIRT3 and mitochondrial biogenesis-related proteins in osteoclasts was affected by daphnetin treatment (Figure S3B,C), the expression of mitochondrial fusion-related protein OPA1, mitochondrial fission-related protein DRP1, and antioxidant enzyme SOD2 in osteoclasts were specifically upregulated by daphnetin treatment (Figure 7H–K).

## 4. Discussion

In the present study, SAMP8 and hindlimb unloading mice were adopted as animal models to evaluate the effects of daphnetin on senile and disuse osteoporosis, respectively. Through micro-CT scanning and H&E staining, we found significant loss of bone mass and deteriorated bone microstructure in both models. Studies have shown that when osteoporosis occurs, cortical bone bears more strength load [27]. In this study, degenerative changes of cortical bone accompanied with that of trabecular bone also occurred, reflecting the comprehensive changes of bone metabolism in SAMP8 and hindlimb unloading mice. Three-point bending tests also showed weakened bone strength in these two models, which were consistent with the results of previous studies [28,29]. It is known that bone strength is reflected in the hardness and toughness of bone. Inorganic minerals determine the density and hardness of bone, while bone organic matter dominated by collagen determines the toughness of bone. Therefore, we detected the content of hydroxyproline, the main representative component of collagen, and the results confirmed the decrease of bone organic matter in both SAMP8 and hindlimb unloading mice. Interestingly, these changes were all effectively reversed by daphnetin administration, indicating that daphnetin has effective osteoprotective effects against senile and disuse osteoporosis.

We further found that the contents of bone remodeling markers related to osteoblastic bone formation and osteoclastic bone resorption in SAMP8 mice were all decreased in bone homogenate, suggesting a low bone turnover status in senile osteoporosis. However, there was a marked increase in serum level of both bone formation- and resorption-related markers in hindlimb unloading mice, indicating a high bone turnover status in disuse osteoporosis. OPG/RANKL pathway, which mediates the molecular correlation between osteoblasts and osteoclasts, plays a vital role in bone remodeling regulation. Up-regulating OPG/RANKL ratio is beneficial to promote osteogenesis and particularly inhibit the formation and activation of osteoclasts [30]. Since osteoporosis is usually accompanied by the decrease of OPG/RANKL, we speculated that the abnormal increase of OPG/RANKL may be an accompanying compensatory effect, and OPG/RANKL may not be the main pathway affecting disuse osteoporosis. Subsequently, the potential mechanisms underlying the protective effects of daphnetin against senile and disuse osteoporosis were further explored. Through activity assays of ALP and TRAP, which are key enzymes of osteogenesis and osteoclastogenesis, as well as ARS and TRAP staining, we found that daphnetin specifically preserved osteoblastic bone formation, but did not reverse the accelerated bone resorption in SAMP8 mice. While in hindlimb unloading mice, daphnetin specifically inhibited osteoclastic bone resorption rather than preserved bone formation. These results further confirmed that the distinct roles of daphnetin in senile and disuse osteoporosis may be dependent on different bone turnover statuses. To our knowledge, this is the first study to explore the osteoprotective effects of daphnetin against senile osteoporosis and disuse osteoporosis, and to reveal that daphnetin alleviates bone loss by distinctly modulating bone formation and bone resorption according to different bone turnover statuses.

It has been demonstrated that oxidative stress and mitochondrial dysfunction are the key pathophysiological characteristics of osteoporosis [31]. Excessive ROS will induce oxidative damage and mitochondrial dysfunction, while mitochondrial dysfunction further leads to more ROS generation. Studies have reported that unregulated accumulation of ROS is a major contributor to aging and age-associated diseases including osteoporosis and other bone diseases [32]. Excessive ROS is known to inhibit osteogenic differentiation and mineralization deposition [33]. Our previous study has demonstrated that SIRT3/SOD2 pathway mediated enhancement of antioxidant capacity and mitochondrial function are essential to osteogenic differentiation [34]. In this study, we consistently found that, the hindered osteogenic differentiation in SAMP8 mice was accompanied with aging-induced oxidative stress and loss of mitochondrial homeostasis in osteoblasts, characterized by increased intracellular ROS level, down-regulation of SIRT3 as well as decreased expressions of mitochondrial biogenesis- and dynamic-related proteins. These data suggest that daphnetin administration may preserve osteogenesis by eliminating excessive ROS of osteoblasts in senile osteoporosis.

To further investigate the mechanisms involved in daphnetin ameliorating oxidative stress in osteoblasts of SAMP8 mice, we evaluated the ability of antioxidant defense system. Because of the vital role of SOD2, CAT, HO-1, and NQO1 as antioxidant enzymes, their expressions, T-AOC, GSH level, and SOD2 activity were determined. There were no significant changes of T-AOC, GSH level, SOD2 activity or its expression in osteoblasts in SAMP8 mice. Although the expression of HO-1 was significantly decreased, daphnetin administration did not reverse this change, but counteracted the excessive ROS generation by up-regulating the expression of enzymatic scavengers CAT and NQO1. Therefore, the role of daphnetin in eliminating oxidative stress of osteoblasts may attribute to inhibiting ROS production rather than enhancing antioxidant capacity and ROS scavenging in senile osteoporosis.

It is known that mitochondrial homeostasis is closely related to the production of mitochondrial ROS. Since daphnetin showed no effect on preserving mitochondrial biogenesis or dynamics of osteoblasts, and ROS produced from NOX participates in regulating various physiological processes including cell differentiation [35], we speculated that the protective effects of daphnetin against oxidative stress may not rely on the improvement of antioxidant defense, or the restoration of mitochondrial homeostasis, but on inhibiting the non-mitochondrial ROS production. Then, we investigated the transcription of NOX family genes, the important source of non-mitochondrial ROS. The transcription of NOX2, the most abundant isoform in osteoblasts, was significantly increased in osteoblasts of SAMP8 mice, while daphnetin administration effectively inhibited the transcription of NOX2, which may result in elimination of ROS. In addition, results of in vitro experiments performed on pre-osteoblast MC3T3-E1 cells also confirmed that daphnetin promoted osteogenic differentiation, and this effect was accompanied with NOX2 inhibition and ROS elimination. Taken together, all these data suggest that daphnetin may preserve bone formation of SAMP8 mice through inhibiting NOX2-mediated ROS production in osteoblast, leading to alleviation of senile osteoporosis.

Accumulating evidence has indicated that intracellular ROS is highly involved in bone homeostasis not only by influencing osteogenesis, but also by intervening osteoclast differentiation [36,37,38,39]. Excessive ROS can down-regulate OPG and simultaneously up-regulate RANKL, then RANKL recruits the linker molecule TRAF6 to the receptor activator of NF-κB (RANK), activates mitogen-activated protein kinase (MAPK) signaling pathway, and promotes nuclear translocation of key transcription factors such as NFATc1 [36], thereby stimulating the expression of downstream osteoclast differentiation marker genes, such as TRAP, Cathepsin K, and MMP9, resulting in activation of osteoclasts and bone resorption, ultimately leading to bone microstructure deterioration and bone density reduction [40,41,42,43]. In this study, we found that daphnetin administration significantly decreased intracellular ROS of osteoclasts, and effectively hindered osteoclast differentiation in hindlimb unloading mice, indicated by inhibited transcription of bone resorption marker genes, decreased expression of NFATC1 and TRAP activity, as well as weakened TRAP staining after daphnetin administration.

SOD2, an important enzyme responsible for reducing ROS in mitochondria, plays an inhibitory role in osteoclastogenesis [44]. In this study, we also found that daphnetin significantly up-regulated SOD2 expression, resulting in enhanced antioxidant capacity and elimination of ROS in osteoclasts of hindlimb unloading mice. In addition, SIRT3 has been confirmed to regulate osteoclastogenesis through activating SOD2 and regulating AMPK-peroxisome proliferator-activated receptor-coactivator 1-beta (PGC-1β) axis [44,45]. Mitochondrial homeostasis including the dynamics of mitochondrial biogenesis, fusion/fission and mitophagy, is also essential for redox state of cells. In this study, we found decreased SIRT3 expression and mitochondrial homeostasis failure in osteoclasts of unloading mice, indicated by significantly decreased expressions of mitochondrial biogenesis- and dynamic-related proteins, while daphnetin administration effectively restored SIRT3 expression and mitochondrial homeostasis of osteoclasts. These results suggest that SIRT3 deficiency and mitochondrial dysfunction in osteoclasts may play an important role in the pathological process of disuse osteoporosis.

Similar to the study performed on osteoblasts of SAMP8 mice, we evaluated the changes of NOX/ROS cascade in osteoclasts of hindlimb unloading mice. It is interesting to note that neither disuse osteoporosis nor daphnetin administration influenced NOX-mediated ROS production in osteoclasts. These results suggest that accompanied with up-regulating SOD2, restoring SIRT3 expression and mitochondrial homeostasis of osteoclasts may be another potential mechanism underlying the protective effects of daphnetin against disuse osteoporosis. Experiments performed on osteoclast precursor RAW264.7 cells further confirmed that daphnetin stimulated the expressions of SOD2 and mitochondrial dynamic-related proteins OPA1 and DRP1 in normal conditions. Perhaps only under pathological conditions can daphnetin show the effects of reducing ROS, preserving SIRT3 and mitochondrial homeostasis. The results of in vitro experiments similarly indicated that daphnetin can enhance antioxidant capacity and is beneficial to quantity- and quality-control of mitochondria in osteoclasts. Although further efforts should be made to illuminate the deep mechanisms underlying the above phenomena, in the current study, we have provided new evidence of the effects of daphnetin on SIRT3/SOD2 pathway and mitochondrial homeostasis of osteoclasts.

In conclusion, we demonstrated that daphnetin alleviated both senile osteoporosis and disuse osteoporosis with different mechanisms. In terms of senile osteoporosis, daphnetin mainly reduced NOX2-mediated ROS production in osteoblasts, resulting in promotion of osteogenic differentiation and bone formation. While in terms of disuse osteoporosis, daphnetin up-regulated SOD2, eliminated ROS, restored SIRT3 expression and mitochondrial homeostasis in osteoclasts, resulting in inhibition of bone resorption. Combined with the results of previous studies, daphnetin has shown a wide range of efficacy and application potential in the prevention and treatment of osteoporosis, although the underlying mechanisms may vary with different types of osteoporosis. In this study, we focused our investigations on the changes of NOX/ROS cascade, SIRT3/SOD2 pathway, and mitochondrial homeostasis involved in osteoprotective effects of daphnetin. However, the deep mechanisms underlying these changes deserve further exploration. Nevertheless, our findings have provided the basis for the application of daphnetin in prevention and treatment of osteoporosis, and also suggest that it may be of great importance to adopt targeted prevention and therapy strategies according to distinct bone turnover statuses of osteoporosis.

## Data Availability

The data presented in this study are available in article.

## Supplementary Materials

The following supporting information can be downloaded at: https://www.mdpi.com/article/10.3390/antiox11122365/s1, Figure S1: Effects of daphnetin on cell viability of MC3T3-E1 and RAW264.7 cells at selected concentrations; Figure S2: Effects of daphnetin on SIRT3, mitochondrial homeostasis and antioxidant capacity of osteoblasts in SAMP8 mice; Figure S3: Effects of daphnetin on NOX cascade in osteoclasts of hindlimb unloading mice, as well as its effects on intracellular ROS content, SIRT3 and mitochondrial biogenesis of RAW264.7 cells; Table S1: Primer sequences used in real-time PCR of this study.
