# Supplementary material for: Daphnetin Alleviates Senile and Disuse Osteoporosis by Distinct Modulations of Bone Formation and Resorption
Source: Antioxidants (Basel). 2022 Nov 29;11(12):2365. doi: 10.3390/antiox11122365 (PMC9774389; doi:10.3390/antiox11122365)
Supplement: Supplementary file 1 [file antioxidants-11-02365-s001.zip › antioxidants-1988143-supplementary.pdf]

Table S1. Primer sequences used in real-time PCR of this study

gene	primer sequence
Runx2	Forward: 5′-TTCTCCAACCCACGAATGCAC-3′<br>Reverse: 5′-CAGGTACGTGTGGTAGTGAGT-3′
Osterix	Forward: 5′-TCCCTACCCAGCGCCCCACCTCT-3′<br>Reverse: 5′-CTGTGAATGGGCTTCTTCCTCAGC-3′
ALP	Forward: 5′-GTTGCCAAGCTGGGAAGAACAC-3′<br>Reverse: 5′-CCCACCCCGCTATTCAAAC-3′
BSP	Forward: 5′-GAATCCACATGCCTATTGC-3′<br>Reverse: 5′-AGAACCCACTGACCCATT-3′
OCN	Forward: 5′-GAACAGACTCCGGCGCTA-3′<br>Reverse: 5′-AGGGAGGATCAAGTCCCG-3′
Col I	Forward: 5′-AACATGACCAAAAACCAAAAGTG-3′<br>Reverse: 5-CATTGTTTCCTGTGTCTTCTGG-3′
OPG	Forward: 5′-CACAGTGAGGAGGAAGACATT-3′<br>Reverse: 5′-GAGAAGAACCCATCTGGACAT-3′
RANKL	Forward: 5′-CAGCATCGCTCTGTTCCTGTA-3′<br>Reverse: 5′-CTGCGTTTTCATGGAGTCTCA-3′
Nfatc1	Forward: 5′-CCAGAAAATAACATGCGAGCC-3′<br>Reverse: 5′-GTGGGATGTGAACTCGGAAG-3′
β-Integrin	Forward: 5′-TTACCCCGTGGACATCTACTA-3′<br>Reverse: 5′-AGTCTTCCATCCAGGGCAATA-3′
MMP9	Forward: 5′-CAGGGAGATGCCCATTTCG-3′<br>Reverse: 5′-GGGCACCATTTGGAGTTTCCA-3′
TRAP	Forward: 5′-CGACCATTGTTAGCCACATACG-3′<br>Reverse: 5′-CACATAGCCCACACCGTTCTC-3′
Cathepsin K	Forward: 5′-GGAAGAAGACTCACCAGAAGC-3′<br>Reverse: 5′-GTCATATAGCCGCCTCCACAG-3′
NOX1	Forward: 5′-TCTCCAGCCTATCTCATCCTGA-3′<br>Reverse: 5′-GCTGCATACATCACTGTCATGTT-3′
NOX2	Forward: 5′-AGTGCGTGTTGCTCGACAA-3′<br>Reverse: 5′-GCGGTGTGCAGTGCTATCAT-3′
NOX3	Forward: 5′-CAAGTGTGTGCTGTAGAGGAC-3′<br>Reverse: 5′-CTATCCCGTAGGCAACGAGTT-3′
NOX4	Forward: 5′-CCTTTTACCTATGTGCCGGAC-3′<br>Reverse: 5′-CATGTGATGTGTAGAGTCTTGCT-3′
NOX5	Forward: 5′-ATGAGTGCCGAGGAGGATG-3′<br>Reverse: 5′-ATCGATGGCAGTGGCTCCAT-3′
DUOX1	Forward: 5′-ACCAGAACATTGCGATGTATGAG-3′

	Reverse:	5′-AGAAATGGACGGTATCCTGGA-3′
DUOX2	Forward:	5′-AAGTTCAAGCAGTACAAGCGAT-3′
	Reverse:	5′-TAGGCACGGTCTGCAAACAG-3′
GAPDH	Forward:	5′-GACTTCAACAGCAACTCCCAC-3′
	Reverse:	5′-TCCACCACCCTGTTGCTGTA-3′

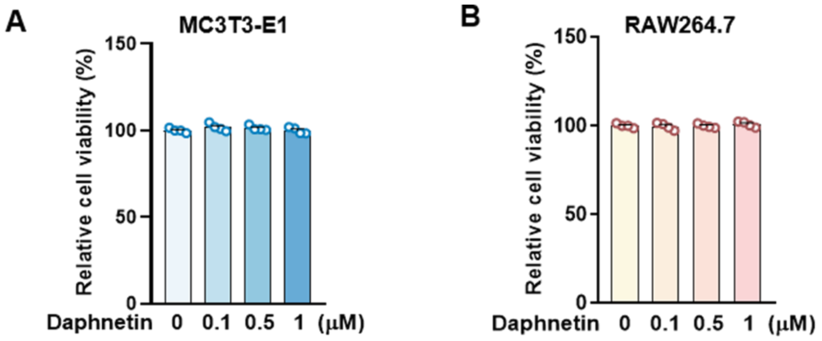

Figure S1. Effects of daphnetin on cell viability of MC3T3-E1 and RAW264.7 cells at selected concentrations. After daphnetin treatment for 48 h without differentiation, cell viability of MC3T3-E1 cells (A) and RAW264.7 cells (B) was determined. Data are presented as the mean ± S.E.M. (N=4).

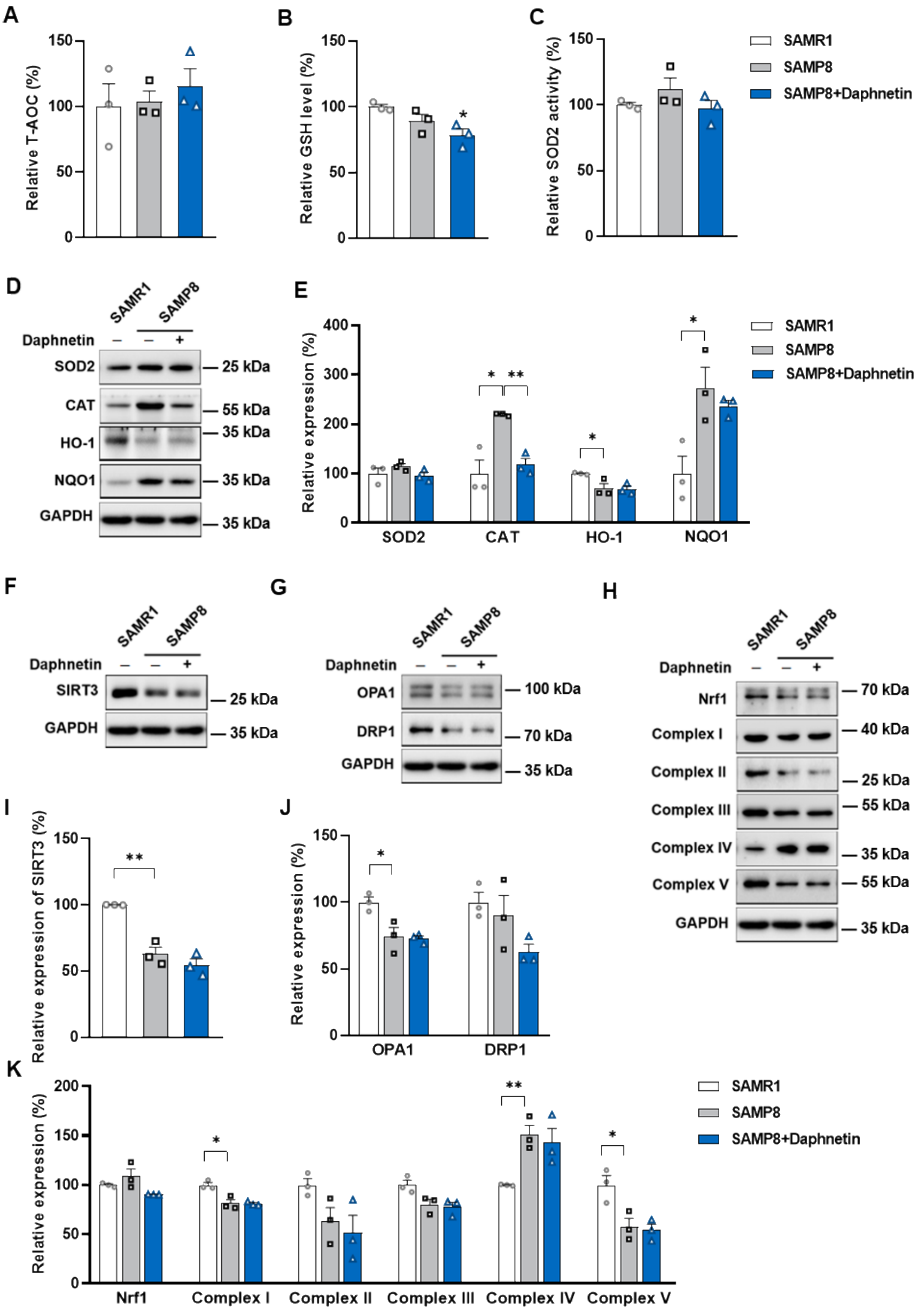

Figure S2. Effects of daphnetin on SIRT3, mitochondrial homeostasis and antioxidant capacity of osteoblasts in SAMP8 mice. Primary osteoblasts were isolated from mice and induced differentiation in vitro. After differentiation for 3 days, Relative T-AOC (A), GSH level (B) and SOD2 activity (C) in cell homogenate were detected, expression of antioxidative enzymes SOD2, CAT, HO-1 and NQO1 were determined by Western blot (D: Western blot image; E: statistical analysis). Expression of SIRT3 was determined by Western blot (F: Western blot image; I: statistical analysis). Expressions of mitochondrial

dynamic-related proteins OPA1 and DRP1 were determined by Western blot (G: Western blot image; J: statistical analysis). Expressions of mitochondrial biogenesis-related proteins were also determined by Western blot (H: Western blot image; K: statistical analysis). Data are presented as the mean ± S.E.M. (N=3); *P<0.05; **P<0.01.

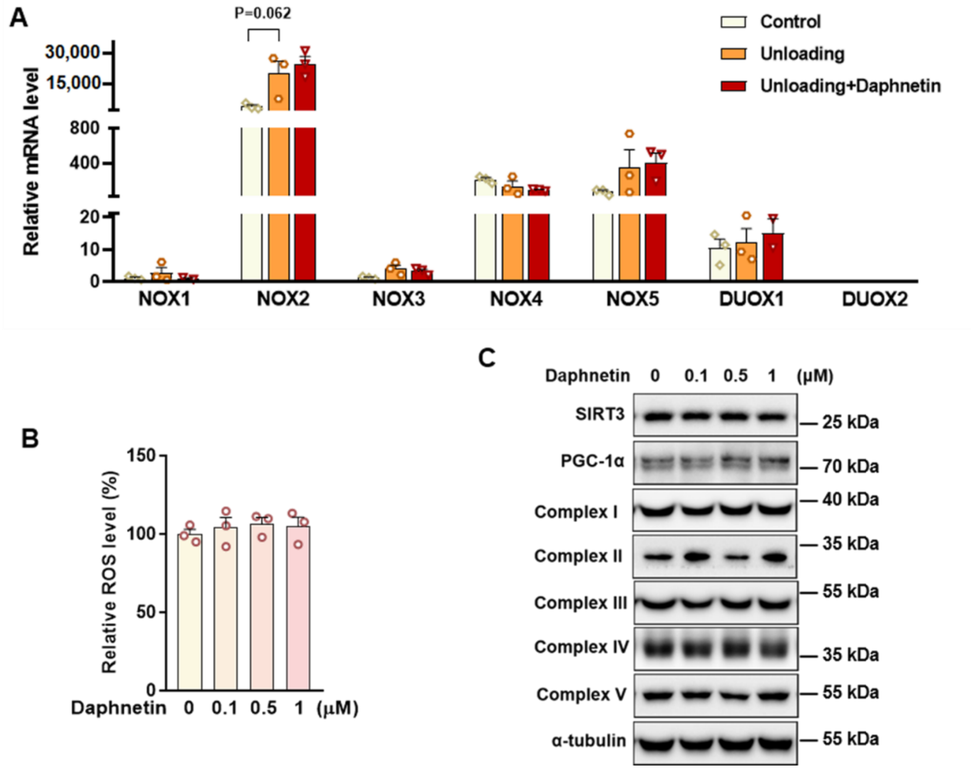

Figure S3. Effects of daphnetin on NOX cascade in osteoclasts of hindlimb unloading mice, as well as its effects on intracellular ROS content, SIRT3 and mitochondrial biogenesis of RAW264.7 cells. Primary osteoclasts were isolated from hindlimb unloading mice and induced differentiation in vitro. (A) After differentiation for 6 days, the transcription of NOX family genes was determined by qRT-PCR. RAW264.7 cells treated with indicated concentrations of daphnetin were induced to differentiate in vitro. (B) After differentiation for 3 days followed by daphnetin treatment for 24 h, intracellular ROS level was determined with DCF probe. (C) After differentiation for 3 days followed by daphnetin treatment for 6 h, expression of SIRT3 and mitochondrial biogenesis-related proteins were determined by Western blot. Data are presented as the mean ± S.E.M. (N=3).
